# A census of α-helical membrane proteins in double-stranded DNA viruses infecting bacteria and archaea

**DOI:** 10.1186/s12859-015-0817-4

**Published:** 2015-11-10

**Authors:** David M. Kristensen, Usman Saeed, Dmitrij Frishman, Eugene V. Koonin

**Affiliations:** National Center for Biotechnology Information, National Library of Medicine, National Institutes of Health, Bethesda, MD USA; Department of Genome Oriented Bioinformatics, Technische Universität München, Wissenschaftzentrum Weihenstephan, Maximus-von-Imhof-Forum 3, D-85354 Freising, Germany; Helmholtz Center Munich - German Research Center for Environmental Health, Institute of Bioinformatics and Systems Biology, Ingolstädter Landstraße 1, D-85764 Neuherberg, Germany; Current address: Department of Biomedical Engineering, University of Iowa, Iowa City, IA USA

**Keywords:** Bacteriophages, Viruses, Comparative genomics, Transmembrane proteins, Membrane proteome

## Abstract

**Background:**

Viruses are the most abundant and genetically diverse biological entities on earth, yet the repertoire of viral proteins remains poorly explored. As the number of sequenced virus genomes grows into the thousands, and the number of viral proteins into the hundreds of thousands, we report a systematic computational analysis of the point of first-contact between viruses and their hosts, namely viral transmembrane (TM) proteins.

**Results:**

The complement of α-helical TM proteins in double-stranded DNA viruses infecting bacteria and archaea reveals large-scale trends that differ from those of their hosts. Viruses typically encode a substantially lower fraction of TM proteins than archaea or bacteria, with the notable exception of viruses with virions containing a lipid component such as a lipid envelope, internal lipid core, or inner membrane vesicle. Compared to bacteriophages, archaeal viruses are substantially enriched in membrane proteins. However, this feature is not always stable throughout the evolution of a viral lineage; for example, TM proteins are not part of the common heritage shared between *Lipothrixviridae* and *Rudiviridae.* In contrast to bacteria and archaea, viruses almost completely lack proteins with complicated membrane topologies composed of more than 4 TM segments, with the few detected exceptions being obvious cases of relatively recent horizontal transfer from the host.

**Conclusions:**

The dramatic differences between the membrane proteomes of cells and viruses stem from the fact that viruses do not depend on essential membranes for energy transformation, ion homeostasis, nutrient transport and signaling.

**Electronic supplementary material:**

The online version of this article (doi:10.1186/s12859-015-0817-4) contains supplementary material, which is available to authorized users.

## Background

All cells are bounded by semi-permeable membranes that consist of lipid bilayers. Eukaryotic cells also possess an elaborate endomembrane system whereas archaea and bacteria, some notable exceptions notwithstanding, lack endomembranes. The integrity of the membrane is essential for the survival of the cell because the membrane maintains gradients of energy, ions and nutrients between the cell and the environment. The membranes of all cells are spanned by diverse integral membrane proteins including energy-dependent transporters, antiporters and channels that are responsible for transport of specific ions or small molecules, and receptors involved in the recognition of various environmental cues. The great majority of integral membrane proteins are anchored in the membrane by hydrophobic transmembrane (TM) helices. The number and orientation of the TM segments determines the topology of the protein in the membrane [1]. Structural characterization of TM proteins is technically challenging and lags far behind the structural analysis of soluble proteins [2–6]. Conversely, however, computational prediction of TM helices from protein sequence is relatively straighforward, and several accurate and fast methods for this task have been developed. These methods have enabled detailed computational analyses of membrane proteomes once multiple complete genomes of diverse organisms have become available for comparative analysis [4, 7].

Comparative genomics has delivered several simple yet powerful insights into the structural variety of TM proteins. The fraction of membrane proteins in cellular proteomes is remarkably constant, roughly 20–25 %, for a broad spectrum of organisms—from bacteria and archaea to complex eukaryotic organisms [8–10]. In other words, the number of TM proteins scales linearly with the total number of genes (and, for bacteria and archaea, with the genome size) and thus fits together with metabolic enzymes in the framework of universal scaling laws of functional classes of genes [11–13]. These findings conform to the model of genome evolution that postulates coupling between the evolutionary trajectories of different gene classes [14] and more specifically imply that evolution of membranes is coupled with the evolution of metabolic networks.

In general, the number of membrane proteins encoded in a genome is inversely proportional to the number of TM helices they contain, with bitopic proteins (those with only a single TM α-helix) being most numerous, and large polytopic proteins (that span the membrane multiple times) much more rare (~10–15 %) [1, 10, 15]. Notable exceptions to this rule include the 6 TM and 12 TM proteins that are over-represented in many multi-cellular organisms, and 7 TM proteins that are extremely abundant in animals [10]. Another general rule is that most TM proteins have both their N- and C-terminal ends facing inwards in the membrane (N_in_-C_in_)—a topology that appears to be strongly preferred in nearly all organisms, with the major exception being the *C. elegans* 7 TM receptors that prefer a N_out_-C_in_ topology [16]—although several instances of proteins with dynamic topologies (temporal and evolutionary) have been described [1].

All of the general conclusions on the abundance, distribution, and structures of TM proteins pertain to cellular life forms. However, the most abundant biological entities on earth are viruses not cells [17]. In a sharp contrast to cells, virus particles are not bounded by closed membranes capable of supporting electrical and chemical gradients. Nevertheless, membranes play important roles in viral reproduction including entrance into the host cells [18–20], replication that often occurs within membranous viral “factories” [21–26], and egress [27, 28]. Some of these interactions between viruses and membranes are facilitated by cellular TM proteins that are hijacked by viruses, but others are encoded in viral genomes. Despite their importance, the current knowledge of the viral membrane proteome is scarse [2, 3, 5, 6, 29]. The carefully annotated SwissProt database [30] contains information about approximately 3000 viral TM proteins, many of them with unknown function. Of these, only a small number of distinct viral proteins have an appreciable coverage of their TM portions by experimentally determined structures. Often, structural studies are instead performed at low resolution and reveal general trends, e.g. the conservation of secondary structure elements in different classes of holins [27]. To the best of our knowledge, no systematic genome-level analysis of viral TM proteins has been performed so far.

Athough the vast majority of the virosphere remains unexplored by sequencing efforts [31–33], the current sequence databases contain over a thousand complete genomes of bacteriophages and archaeal viruses, together encoding >10^5^ proteins. Notwithstanding the typical fast evolution of viral genomes, many of these proteins have detectable homologs in other viruses [34]. Recently, the evolutionary conservation of protein-coding genes among bacterial and archaeal viruses has been captured in the collection of Prokaryotic virus Orthologous Groups (POGs) that currently includes >4500 gene families [35, 36]. The POGs include orthologous genes from DNA and RNA viruses that infect bacteria or archaea, although nearly 90 % of the genomes and 97 % of the conserved proteins are from the large double-stranded DNA (dsDNA) viruses, which mostly represent the tailed bacteriophages of the order *Caudovirales*. This heavy bias towards the tailed bacteriophages appears to reflect the situation in nature because these viruses are indeed the most abundant genome-containing entities on earth [37], outnumbering cells by about ten-to-one [38, 39].

Here we describe the use of POGs combined with methods for TM prediction to generate a comprehensive genome-scale census of α-helical TM proteins encoded by dsDNA viruses infecting bacteria and archaea, and compare the differences between this complement and that of their cellular hosts. We find that viruses show the expected dependence between the number of TM proteins and the total number of genes (genome size), but typically encode a much smaller fraction of TM proteins than bacteria and archaea and also show a much greater variance of that fraction. Furthermore, viruses almost exclusively lack more complicated membrane topologies with more than 4 TM segments, with a few exceptions that appear to represent proteins recently acquired from their hosts. These findings imply that viruses do not follow the general scaling laws for functional classes of genes that appear to hold for all cellular organisms.

## Results

### Overall TM complement of dsDNA prokaryotic viruses

Focusing on the >900 genomes available for the extensively studied class of dsDNA viruses that infect bacteria and archaea, POGs were constructed and TM predictions were made as described in the Methods. These represent virus groups such as *Caudovirales* (~86 %), *Tectiviridae* (~1 %) and several other families of viruses infecting bacteria and archaea. (Additional file 1: Table S1). Overall, a typical dsDNA virus genome consists of ~80–100 proteins, although this number varies by 2 orders of magnitude from the tiny 2.4-kbp *Leuconostoc* phage *L5* with only 5 proteins, up to the nearly 500-kbp genome of *Bacillus* phage G with >700 proteins. Like genome size, the proportion of proteins per genome that are conserved in POG gene families is highly variable, from none of the 5 proteins in *Leuconostoc* phage *L5* or other poorly characterized viruses, up to 100 % in several well-characterized *Staphylococcus* and *Mycobacterium* phages. On average, a typical genome contains 50–60 conserved proteins that make up ~60 % of its protein complement [35].

**Fig. 1:**
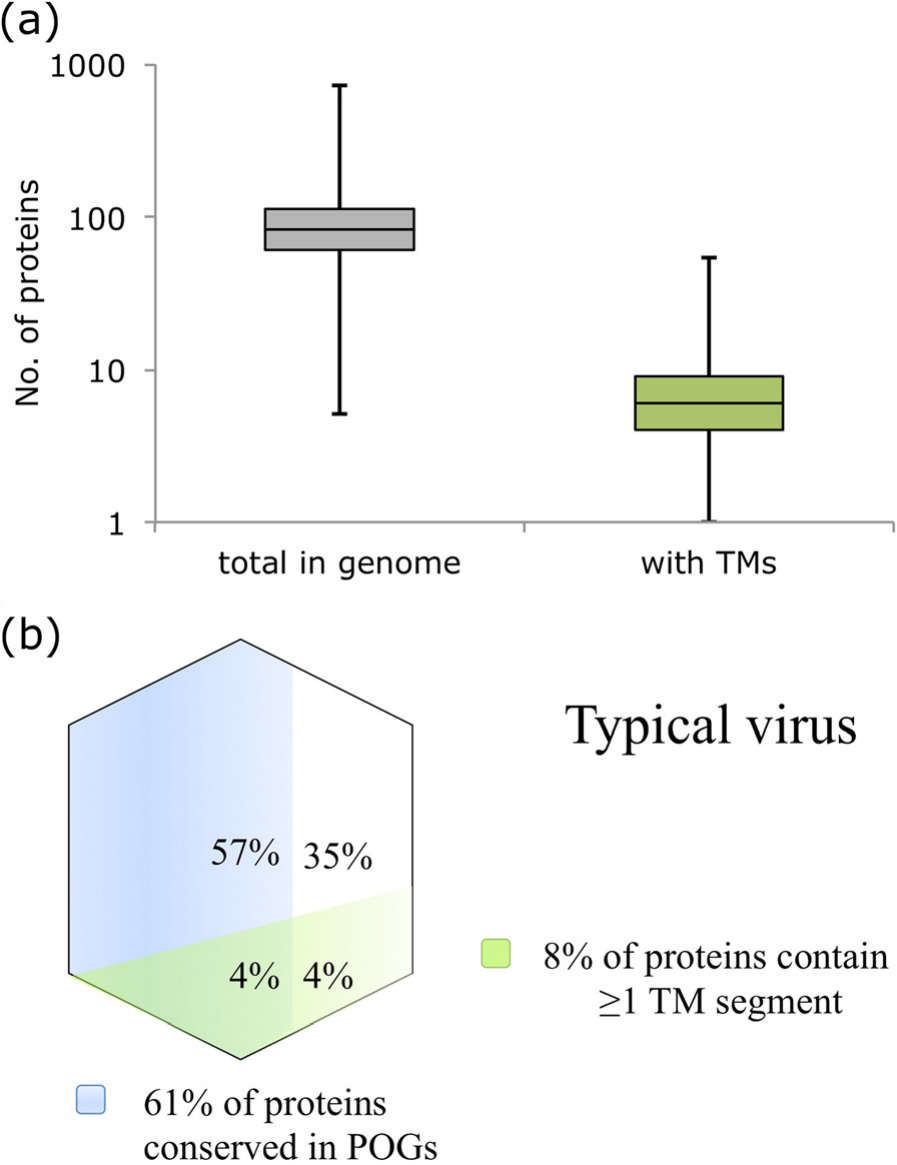
Overall distributions of proteins in dsDNA genomes of viruses infecting bacteria and archaea. **a** Boxplot showing the overall ranges of the number of proteins in each genome overall, vs. TM proteins. **b** Venn diagram showing the typical virus complement (average percentages) of TM/non-TM proteins and proteins conserved/not conserved in POGs

These viral genomes typically encode few TM proteins, <10 % for most viruses (Fig. 1a). Only about 15 % of the analyzed viral genomes encode >10 % (and up to 41 %) of TM proteins. These TM-rich viruses include the *Enterobacteria* phage PR group of the family *Tectiviridae* and several archaeal viruses of the families *Fuselloviridae*, *Rudiviridae*, *Plasmaviridae*, *Globuloviridae*, *Ampullaviridae* and other groups (Additional file 1: Table S1). At the other end of the spectrum, the only virus not found to contain at least one TM region is the tiny Mycoplasma phage P1 with only 11 proteins altogether. The proportion of TM proteins that are homologous between viruses (as judged by representation in POGs) is roughly the same as for non-TM proteins (Fig. 1b).

**Fig. 2:**
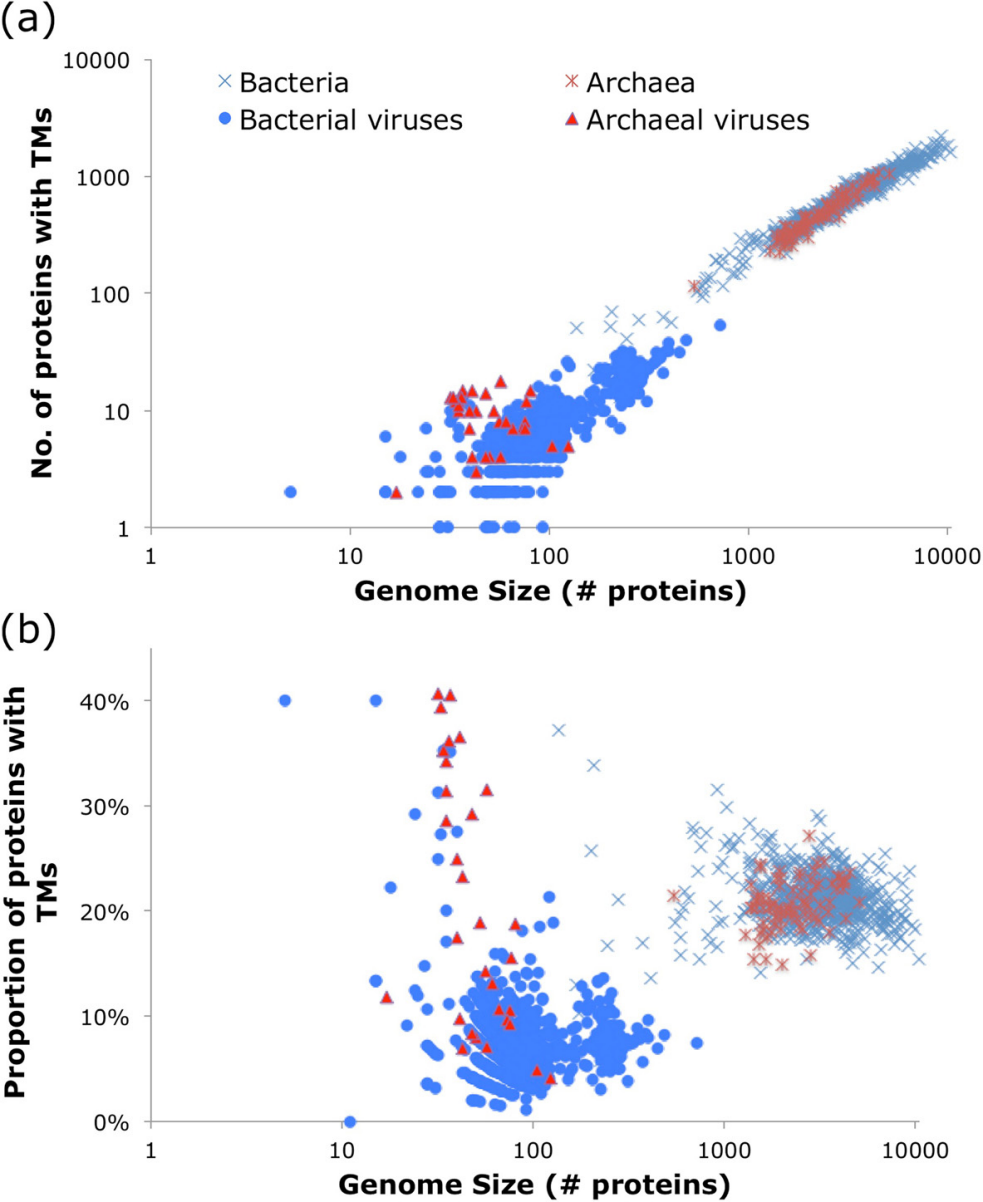
TM proteins and genome size. The plots of (**a**) number and (**b**) proportion of TM proteins vs. genome size

The number of TM proteins encoded in viral genomes scales roughly linearly with the genome size although the relative deviation away from the linear trend line is much greater than in bacterial and archaeal genomes (Fig. 2a). The difference between the distribution of TM proteins among viruses compared to that among prokaryotes becomes more apparent when the fraction of TM proteins is plotted against the genome size (Fig. 2b). In agreement with previous observations, the fractions of TM proteins in bacteria and archaea do not depend on the genome size and vary within the range from 15 to 30 %, with most genomes encoding between 20 and 25 % membrane proteins; the few outliers with TM proportion >30 % are highly degraded intracellular parasites that encode only several hundred proteins (Fig. 2b). Among viruses, the variation in the fraction of TM proteins is much greater, with the majority being <10 % and thus well separated from bacteria and archaea, but a minority encoding a large fraction of TM proteins, within the microbial range and higher (Fig. 2b). Noticeably, the high TM content was found only in viruses with small and moderate (below average) genome size, conceivably due to the large repertoires of non-TM proteins, such as regulators of host cell transcription, translation, and other metabolic activities, in viruses with larger genomes. Presumably, the typically low but widely varying TM content among viruses has to do with the removal of the functional constraints that dictate the nearly constant proportion of TM proteins in cellular life forms.

Although TM proteins in viruses differ in several respects from those in cellular organisms, many characteristics remain similar. Specifically, the amino acid distribution does not differ significantly (Chi-Squared test), the “positive-inside” rule of TM topology is still observed, the characteristic length of TM segments is the same at roughly 20aa, and a very low proportion of signal peptides is found [40] (<5 %).

### TM content and topology in viral and microbial proteins

**Fig. 3:**
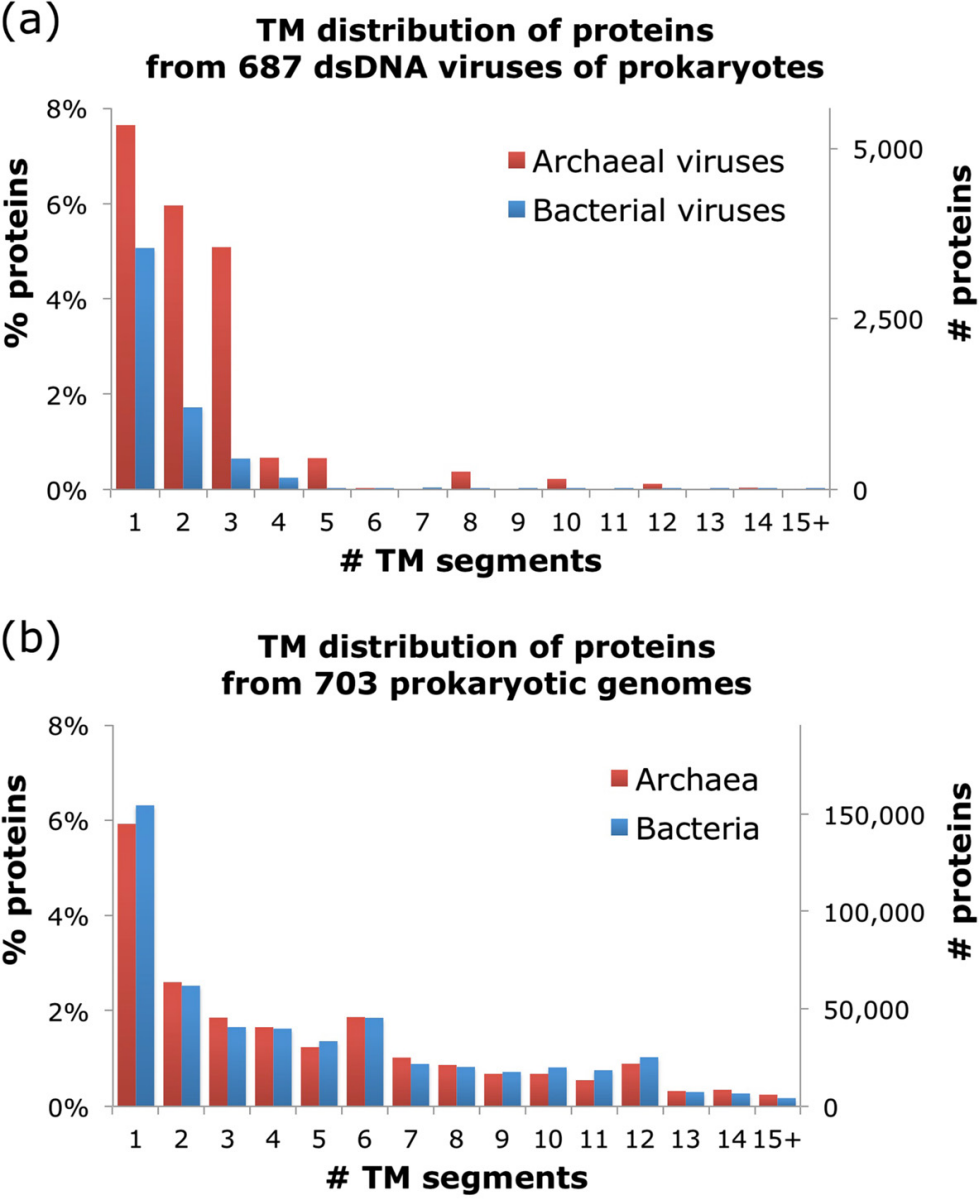
TM topology. Number and proportion of TM segments in (**a**) virus and (**b**) host genomes

The distributions of membrane proteins by the number of TM segments in representative sets of prokaryotic and viral genomes show striking, highly significant differences (*p*-value < 1e-300 by Chi-Squared test and <2e-16 by Mann-Whitney-Wilcoxon test) whereas the archaeal and bacterial distributions are indistinguishable (Fig. 3). Although in both viruses and cellular organisms the most prevalent group includes single-TM proteins, bacteria and archaea have a heavy tail of multi-TM proteins, with second and third peaks at 6 TM and 12 TM, respectively. In a sharp contrast, proteins with 5 or more TM segments are extremely rare in viruses (compare Fig. 3a and b).

Moreover, those virus proteins that are predicted to contain >5 TM often have more homologs among bacteria than among other viruses (Additional file 1: Table S2), suggestive of a relatively recent horizontal transfer of the respective gene from bacteria (even if the function of the protein changed in the viral context). For example, several photosystem components found in cyanophages [41] with 6–7 TM segments (reaction center D1 and D2, along with other components with fewer TM segments (e.g. plastoquinol terminal oxidase with 1 TM), have been recently borrowed from their cellular hosts. Another example is nicotinamide mononucleotide transporter with 7 TM segments that is found in only 6 tailed bacteriophages infecting several bacterial genera but is widely represented in bacterial genomes. Yet another class of functions includes o-antigen conversion proteins with 2, 4, and 11 segments that are found in many more bacteria than viruses. Conversely, proteins with <5 TM segments typically include functions such as holins, virion components (tail tape measure, end-filament, tail, baseplate, and related functions such as head-to-tail joining proteins, DNA packaging, DNA delivery, scaffold, lysis cytotoxic factors, Na/K ATPase and more). A comprehensive analysis of the functions of viral TM proteins is hampered by the paucity of experimental data for the great majority of these proteins and the lack of functionally characterized homologs.

Among the majority of virus TM protein topologies, roughly equal proportions of proteins were observed to be conserved vs. not conserved in POGs (data not shown). An identical analysis was performed on prophages integrated into host chromosomes, with results qualitatively similar to their lytic counterparts: about 10 % of the prophage proteins contain at least one TM region, and a similar depletion of proteins with more than a single TM is observed (data not shown).

Another notable feature of the viral TM protein distribution is the obvious, highly significant difference between the viruses infecting bacteria vs. archaea (<2e-16 by Mann-Whitney-Wilcoxon test) which is examined in greater detail below.

### Robustness of TM prediction

To ascertain that the difference in the number of TM observed in viruses vs. cellular organisms is a biological effect rather than a mere technical artifact, the analysis was repeated using the MEMSAT3 program (see Methods) for the 5 virus genomes with the highest prevalence of TM proteins. In this dataset, 81 % of the results were identical to those of the global analysis using Phobius, and among the cases that were not, 91 % involved a difference of only a single TM segment, with the other 9 % involving only two segments. Thus, both Phobius and MEMSAT3 confirm the overall lack of more complicated TM topologies in viruses.

As another assessment of the accuracy of TM prediction, we analyzed the consistency of predictions among proteins in the same POG (see Additional file 2: Table S2). For 94 % of the POGs, the same prediction was obtained for all the proteins (99 % of proteins with multiple TM segments). When orthologs differed in their TM assignments, more than 50 % did so only by a single TM segment, whereas another 25 % differed by two TM. Greater differences were observed for a small number of proteins noticed previously, in particular for viruses [1]. These differences mostly involve poorly alignable protein regions: for instance, in holins that are small hydrophobic proteins, different numbers of TM segments are sometimes predicted in different regions of the sequence, and it remains unclear which of these reflect articfacts and which are biologically relevant differences. Despite these uncertainties, the overall excellent agreement of the TM prediction results among different proteins within the same POG provides confidence in the validity of the trends observed in this study.

**Fig. 4:**
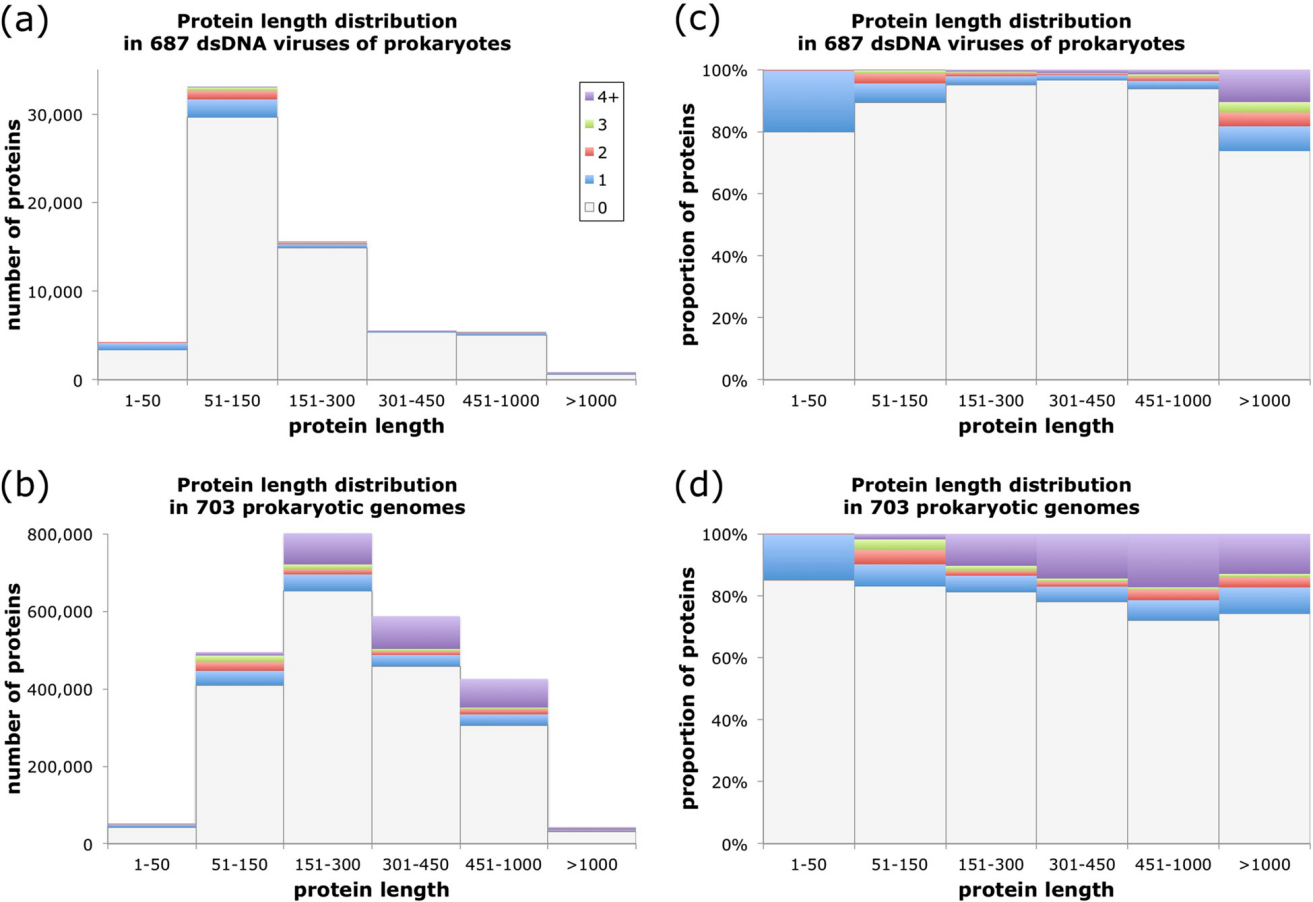
Protein length and TM segments. Protein length distribution according to number of TM segments (0 through ≥4) in (**a**) virus and (**b**) cellular genomes, and proportion of proteins in each protein length bin for (**c**) virus and (**d**) cellular genomes. Protein length values are in units of number of amino acids

To further assess the accuracy of the TM topology assignments and verify that the small number of TM segments in virus proteins is not a consequence of the small characteristic size of viral proteins, we analyzed the dependence of TM predictions on protein length. Figure 4 shows that despite the typical difference in protein length—easily visible in the shift between the protein length distributions of viruses (Figs. 4a) and cells (Fig. 4b)—this difference cannot be solely responsible for the dissimilarity in TM topologies. As the corresponding proportion plots in Fig. 4c and d demonstrate, at the same length, cellular organisms display a greater fraction of polytopic proteins containing higher numbers of TM segments than viruses.

### TM protein complements in different types of viruses

**Fig. 5:**
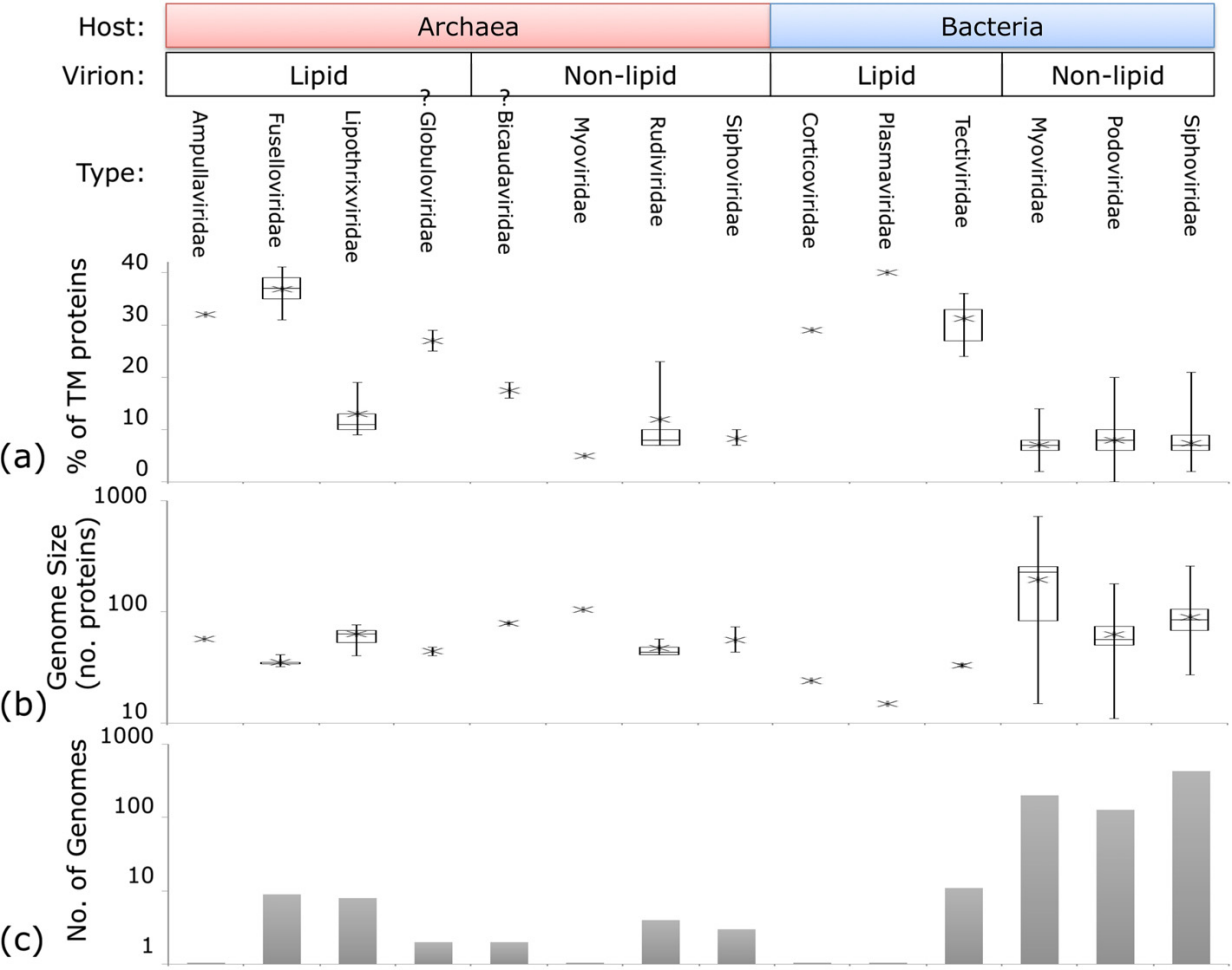
Virus types. For each virus family, the (**a**) proportion of TM proteins, (**b**) genome size, and (**c**) number of genomes is shown. Virus families are also labelled by host domain and lipid/non-lipid association of the virion. The star represents the average value, with whiskers showing minimum and maximum (if family has more than one genome), and boxplots shown for families containing at least 4 genomes

To investigate the TM complements in different types of viruses, the characteristics of each virus are described in terms of three categories: the virus taxonomic family, the domain of the host (either Bacteria or Archaea), and whether the virion particle is directly associated with lipids (having an envelope, an internal lipid core, or an inner membrane vesicle) (see Methods and Additional file 3: Table S3). With the sole exception of the archaeal virus family *Lipothrixviridae*, all viruses with a lipid-associated virion encompass higher fractions of TM proteins than viruses with a lipid-less virion (Fig. 5a). Among viruses with lipid-containing virions, the fraction of TM proteins range from 24 % at the low end in *Bacillus thuringiensis bacteriophage Bam35c* (*Tectiviridae*) to 41 % in two *Sulfolobus* spindle-shaped viruses (*Fuselloviridae*). The lipid-less viruses have a proportion of TM proteins that is typically less than 10 %, with only 3 viruses having >20 %, up to a maximum of 23 % in *Sulfolobus turreted icosahedral virus* of *Rudiviridae*. The TM proportion of *Globuloviridae* (only tentatively assigned as “lipid-associated”) falls in the same range as other lipid-associated viruses, and that of *Bicaudaviridae* (tentatively assigned as “non-lipid-associated”) falls in the same range as other lipid-less viruses.

**Fig. 6:**
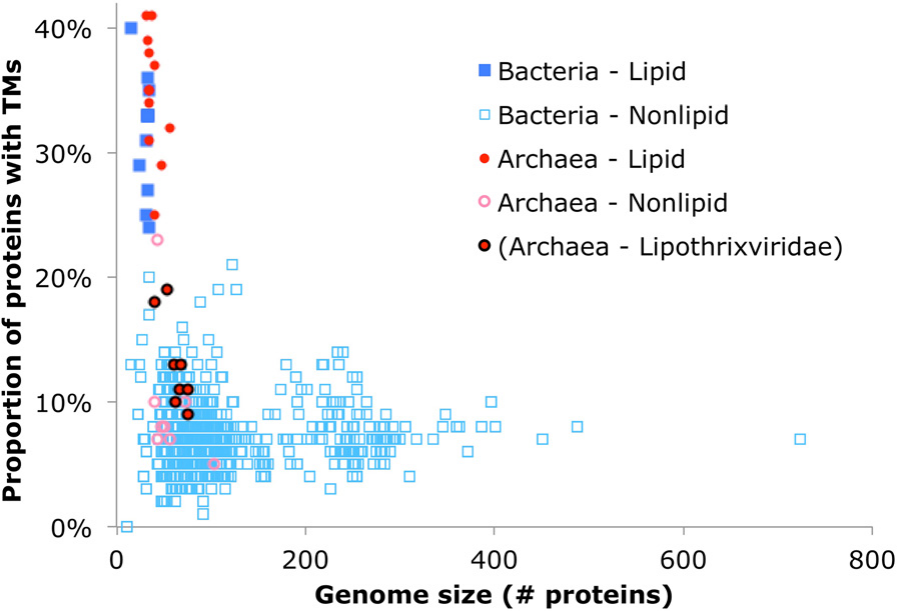
TM proportion and genome size, by virus category. Proportion of TM proteins vs. genome size, colored according to host domain and virion lipid association. Datapoints belonging to the virus family *Lipothrixviridae* outlined in black

The data in Fig. 5 (a, b, and c) demonstrate that it is the presence of lipids in association with the virion, not genome size or host domain, that is most closely correlated with the proportion of TM proteins. For instance, *Ampullaviridae, Fuselloviridae*, *Rudiviridae* and the archaeal *Siphoviridae* viruses all share the characteristics of infecting an archaeal host and having a small genome size (<100 proteins). However, the former two families form lipid-associated virions and have a high proportion of TM proteins, whereas the latter two have lipid-less virions and a correspondingly low proportion of TM proteins (Fig. 5). The sharp distinction between viruses with lipid-containing and those with lipid-less virions is even more apparent in Fig. 6 where, with the exception of *Lipothrixviridae,* all viruses with lipid-associated virions—regardless of genome size and host range—show perfect separation from the viruses with lipid-less virions in terms of the proportion of TM proteins. The nonparametric Mann-Whitney-Wilcoxon test confirms the independence of the lipid vs. non-lipid-associated populations with p < 0.005, and there is a strong positive correlation between the proportion of TM proteins and lipid association (correlation coefficient 0.776), compared to the weaker correlation of the TM protein proportion with host domain (bacteria vs. archaea) (0.467), and the weak negative correlation with genome size (number of proteins) (−0.18).

**Fig. 7:**
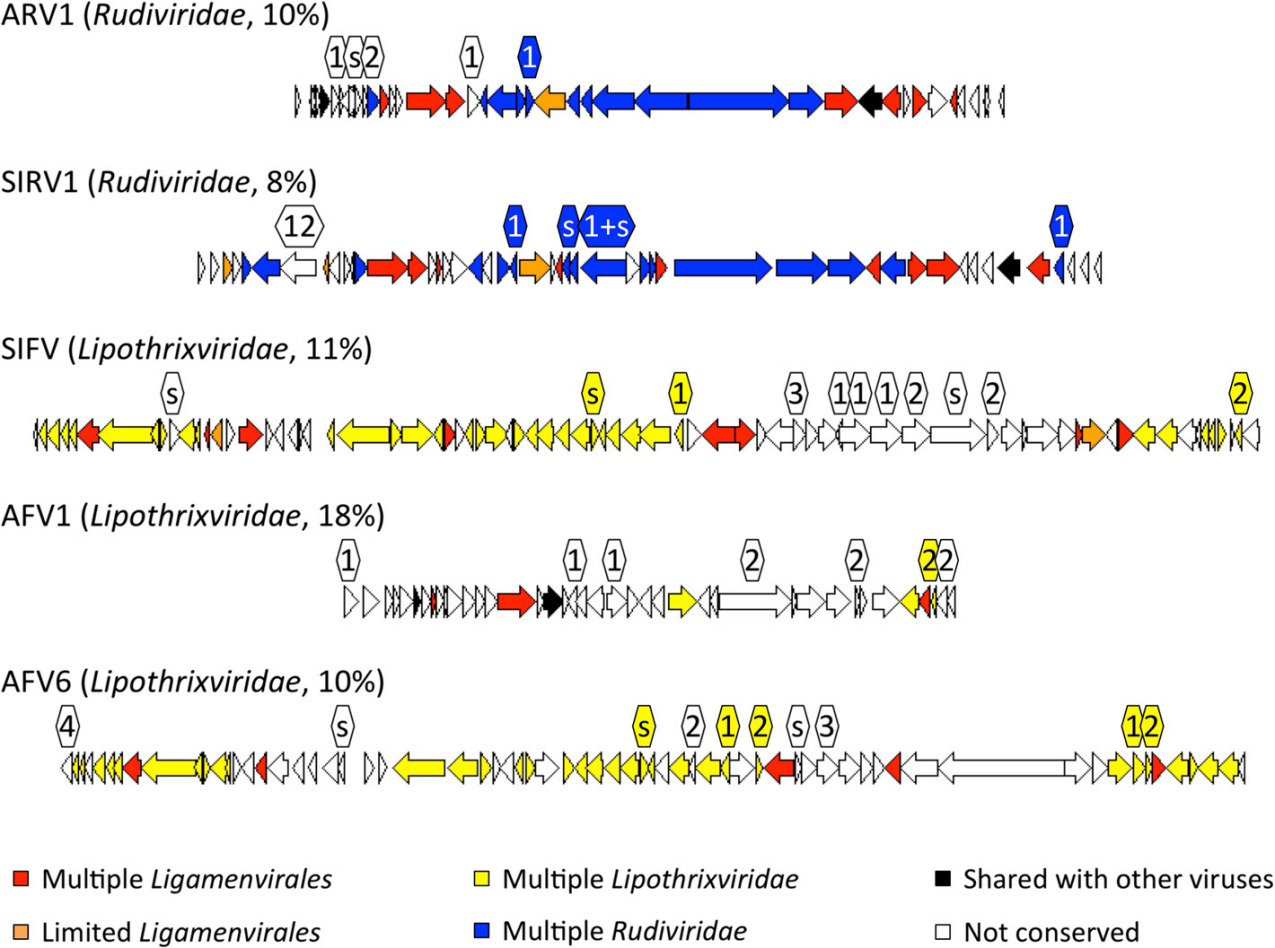
Genes shared in viruses of *Lipothrixviridae* and *Rudiviridae.* Genes shared between *Acidianus rod-shaped virus 1* (ARV1, *Rudiviridae*, with 10 % of its proteins containing TM segments), *Sulfolobus islandicus rod-shaped virus 1* (SIRV1, *Rudiviridae*, with 8 % of TM proteins), *Sulfolobus islandicus filamentous virus* (SIFV, *Lipothrixviridae*, with 11 % of TM proteins), *Acidianus filamentous virus 1* (AFV1, *Lipothrixviridae*, with 18 % of TM proteins), and *Acidianus filamentous virus 6* (AFV6, *Lipothrixviridae*, with 10 % of TM proteins). Genes shared by multiple genomes within both *Lipothrixviridae* and *Rudiviridae* are colored red; or orange if only present in a single genome of either family; genes found in multiple representitives of the 8 genomes of *Lipothrixviridae* (available as of the last update of the POGs, although only 3 representatives shown here) colored yellow; or found in multiple representitives of the 4 genomes or *Rudiviridae* (although only 2 representatives shown here) colored blue; genes shared only with viruses from other families colored black; while genes not conserved in POGs colored white. Numbers above a gene represent the number of TM segments that it contains, with sequences containing a signal sequence designated by “s”, and colored in the same way as the genes

The viruses of the family *Lipothrixviridae* are an exception to the simple rule under which the presence of lipids in virions dictates the content of TM proteins. Both the number and the proportion of TM proteins in the lipothrixvirus genomes are much lower than those of the other families of archaeal lipid-asscoiated viruses, *Ampullaviridae* and *Fuselloviridae*. The viruses of the families *Lipothrixviridae* and *Rudiviridae* share several homologous proteins and similar genome architectures and have been included in the single order *Ligamenvirales* [42]. Thus, it appears possible that the lipid association of the *Lipothrixviridae* virion is a relatively recent innovation that evolved after the divergence from the common ancestor with *Rudiviridae*; alternatively, rudiviruses might have lost the membrane association. To explore these possibilities, we compared the genome organization between *Lipothrixviridae* and *Rudiviridae*. In agreement with previous reports, the definition of conserved genes based on shared POGs that was employed here indicate considerable conservation between the two virus families (Fig. 7). Notably, however, the TM proteins are not part of this common heritage. Moreover, many of the genes encoding these largely uncharacterized proteins are not conserved even within their respective families *Lipothrixviridae* or *Rudiviridae* (less than a third of TM proteins in *Lipothrixviridae* are conserved in other virus genomes). Thus, it appears likely that the lipid-association of *Lipothrixviridae* virions is not a stable feature of this group, but rather is in evolutionary flux and is likely a recently acquired feature (a common feature in prokaryotes and certainly not unexpected in viruses [43]).

## Conclusions

Viral transmembrane proteins and their roles in virus reproduction and virus-host interaction are extremely poorly studied. The repertoires of TM proteins in viruses infecting prokaryotes radically differ from those in archaeal and bacterial cells. Unlike cellular organisms in which TM proteins represent a nearly constant fraction in the range of 20 to 25 %, the variation in the fraction of TM proteins in viruses is much broader. The majority of viruses posses less than 10 % TM proteins but a substantial minority are TM-rich, with over 40 % TM proteins in some. Furthermore, there is only weak scaling of the number of TM proteins with the genome size of viruses as opposed to the near perfect proportionality in bacteria and archaea. Finally, viral membrane proteomes consist predominantly of proteins with a single TM, in contrast to the membrane proteomes of bacteria and archaea that are rich in TM proteins with multiple TM segments, in particular 6-TM and 12-TM proteins.

These dramatic differences between the membrane proteomes of viruses and cellular organisms seem to have a straightforward explanation in the completely different roles played by membranes in the reproduction of cells and viruses. In cellular life forms, the membranes perform a well-defined suite of essential functions that have to do with energy transformation, ion homeostasis, nutrient transport and signaling. This suite of essential, universal functions dictates the near constancy of the TM protein fraction in bacterial and archaeal genomes. Moreover, the nearly identical distributions of membrane proteins by the number of TM segments in bacteria and archaea imply even more detailed, universal functional constraints. None of these functions are relevant in most viruses, and on the few occasions when such activities are performed by viral proteins, they are involved in virus-host interaction rather than the central process of virus reproduction. Due to the lack of universal, essential membrane functions, viruses show a broad range in the fraction of TM proteins, with most viruses encoding only a few but some showing a greater proportion of TM protein than cellular life forms. A more general implication of these findings is that viruses do not typically obey the universal scaling laws for functional classes of genes that seem to apply in all cellular life forms [11–13].

Viral TM proteins can be involved in two classes of functions: first, virion structure formation, and second, modification of membranes in the infected cell. Clearly, the TM proteins can contribute to virion structure only in those viruses that possess some form of lipid membranes. The observation that all viruses with high TM content also contain lipids in their particles indicates that the involvement of TM proteins in virion morphogenesis is indeed substantial in these cases. Most of the viral membrane proteins supporting virion structure have simple, single-TM architectures. A notable feature that became apparent in the course of this work is the higher prevalence of lipid-containing, TM-rich viruses in archaea compared to bacteria. Whether the greater involvement of membranes in virion structure of archaeal viruses is an adaptation to extreme conditions or reflects other aspects of their lifestyle remains to be determined. The roles of viral TM proteins in host membrane modification are poorly understood but generally fit within the paradigm of virus-host interaction. A well-characterized case are the cyanophages that carry TM proteins of photosystems that boost nutrient production in the infected cyanobacteria [44].

It is our hope that the present census of TM proteins in viruses of bacteria and archaea facilitates experimental characterization of the role of membranes in viral reproduction [1–6]. A comparative genomic census of membrane proteins in the numerous and diverse viruses infecting eukaryotes will similarly help in revealing universal and host-specific aspects of virus evolution.

## Methods

### Dataset

The genomes of dsDNA viruses that infect bacteria and archaea were downloaded from the RefSeq and Nucleotide databases of NCBI. Clusters of orthologous groups within these prokaryotic viruses (POGs) were constructed as described previously [35] using the standard methodology [45–47].

For overall statistics (e.g., Fig. 1 and 2), all 903 genomes of dsDNA viruses were used (Additional file 1: Table S1). For the direct comparisons between viral and cellular organisms (e.g., Fig. 3), a representative subset of 687 viruses was chosen to reduce redundancy bias such that for groups of viruses that share at least 90 % of their genes, only one representative genome (chosen randomly) was used to represent the lineage and the rest discarded. A dataset of >700 bacterial and archaeal genomes were also downloaded from the RefSeq database at NCBI, with redundancy bias reduced by picking a single representative (largest genome) from each genus. Proviruses that were integrated into cellular genomes were identified by the Phage_Finder program [48], using version 2.1 and default parameters.

For every virus genome, the taxonomic family of the virus and the domain of the host cell (Bacteria vs. Archaea) was obtained from the Taxonomy database of NCBI. The lipid association of the virion particle was manually assigned to each virus family based on the description of that virus in the ViralZone database [49]. Specifically, virions that are enveloped (*Ampullaviridae*, *Fuselloviridae*, *Lipothrixviridae*, and *Plasmaviridae*), have an internal lipid core (*Corticoviridae*) or enclose an inner membrane vessicle (*Tectiviridae*) were labelled as lipid-associated, whereas virions lacking any of these (*Rudiviridae* and the tailed viruses *Myoviridae*, *Podoviridae*, and *Siphoviridae*) were labelled as non-lipid-associated. The status of *Globuloviridae* is indeterminate, but since it is considered to be presumably enveloped, was tentatively included along with lipid-associated virions. In a similar fashion, *Bicaudaviridae* was tentatively assigned to non-lipid-associated virions.

### Prediction of protein transmembrane topology

Phobius [50] version 1.01 was run with default parameters on each virus and cellular genome, after removing nonstandard amino acids (ambiguity codes). Phobius is an accurate hidden Markov model-based predictor of TM topology (and signal peptides) that is well suited for high-throughput computational screening of complete proteomes due to its ability to distinguish between TM vs. non-TM proteins, its high speed, and lack of a requirement to first build a multiple sequence alignment. The latter is a particularly useful property among virus proteins that do not have many homologs available in the protein sequence databases. Although Phobius was primarily trained on cellular TM proteins, we did not observe a statistically significant difference in the amino acid composition of 13,117 and 43 experimentally confirmed **α**-helical TM regions from cellular and viral proteins, respectively, obtained from the TOPDB database [51] (data not shown), which demonstrates that the physico-chemical properties of these proteins are similar.

To confirm the TM topology predicted by Phobius, MEMSAT3 (version 3.0) was also used and the results compared. Unlike Phobius, MEMSAT3 does not predict whether a protein sequence belongs to a TM protein or a non-TM protein, but instead assumes *a priori* that every input protein contains at least one TM. Among TM-containing proteins, it may offer greater accuracy in predicting the exact number of TM regions that are present [7, 52]. MEMSAT3 also requires a multiple sequence alignment/profile to be constructed for every input sequence. Therefore, MEMSAT3 was run with default parameters on those proteins predicted by Phobius to contain at least one TM region, and the number of TM segments recorded. To build the multiple sequence alignment/profile used by MEMSAT3, the PSI-BLAST [53] search for homologs was performed with the database of all proteins found in completely sequenced genomes of the viruses that infect bacteria or archaea.

Signal peptides were predicted using the SignalP program [54], version 4.1, with all three training models tested.

### Statistical procedures

Average numbers were used in the description of a “typical virus” complement of Fig. 1, with no qualitative difference observed between use of median vs. average.

The virus specificity was measured for each POG similar to the description in Kristensen et al. [35]. Briefly, the Viral Quotient (VQ) is the ratio of the frequency of matches to viral genomes divided by the sum of both the viral and cellular genomes matches were found in. The frequency of homologs appearing in easily-detected prophage regions (identified with the Phage_Finder program [48]) was added to both the viral and cellular fractions. Thus, proteins with VQ = 1.0 are those only observed in lytic viruses, proteins with VQ higher than 0.5 represent predominantly phage- and prophage-related proteins (having more homologs in viruses than in non-prophage regions of bacterial chromosomes), and proteins with VQ < 0.5 represent predominantly cellular proteins (with more homologs in cellular organisms than viruses).

All statistical tests (Chi-squared and nonparametric Mann-Whitney-Wilcoxon tests, and Pearson correlation) were performed using standard methodology.

## Availability of supporting data

The data set(s) supporting the results of this article are included within the article (and its additional files).

## Additional files

Additional file 1: Table S1. Phage dataset. Counts for the numbers of proteins, TM proteins, and proteins conserved in POGs for each genome in the dataset. (XLSX 325 kb) Additional file 2: Table S2. Conserved TM POGs. Descriptive data for each conserved TM POG, such as viral specificity and number of TM segments. (XLSX 1213 kb) Additional file 3: Table S3. Virus categories. Description of virus categories and data shown in Figs. 5 and 6. (XLSX 49 kb)

## Abbreviations

TM: Transmembrane
dsDNA: Double-stranded DNA
POG: Prokaryotic virus Orthologous Groups
VQ: Viral Quotient
